# Supplementary material for: Patterns of Intron Gain and Loss in Fungi
Source: PLoS Biol. 2004 Nov 30;2(12):e422. doi: 10.1371/journal.pbio.0020422 (PMC532390; doi:10.1371/journal.pbio.0020422)
Supplement: Table S1 — Also available at http://genes.mit.edu/NielsenEtAl/. (4.3 MB ZIP). [file pbio.0020422.st001.zip › NielsenEtAl/html/888.html]

AN5686.1.NCU01204.1.MG08078.1.FG07443.1


```
 CLUSTAL W (1.82) Multiple Sequence Alignments - Introns Inserted


Sequence 1: MG08078.1	161 aa
Sequence 2: FG07443.1	161 aa
Sequence 3: NCU01204.1	161 aa
Sequence 4: AN5686.1	161 aa
Alignment Length: 161 aa
Number Identitical Residues: 99 aa
Alignment Score (without introns) 4253


MG08078.1 	MDRIKE0KMNQLRLEADESNAKVEELQSKVKLLEQENLSKEQEITSLQHKNGVLEGELEK
NCU01204.1	MDRIKE0KMNQLRLEADEASAKVEELQSKIKVLEQENLQKEQEITSLSHKNSVLESEVEK
FG07443.1 	MDRIKE0KMNQLRLEADEAAGKVEELQTKVKVLEQENLSKEQEITSLQHKNNLLEGEVEK
AN5686.1  	MDRIKE0KMNALRLEADEAHEKNEELKARVKTLEQENLAKEQEITSLTHRNQLLEAEVEK
          	****** *** *******:  * ***::::* ****** ******** *:* :**.*:**

MG08078.1 	LEENVKNLKKMADENVSAGTQNENLARRLQLLEDEAEEADKTLREANEK2LRQTDVKAGH
NCU01204.1	LETQVKEFKSAAEDGAQQGTQNETLTRRLQLLEEEAEEADKTLRETNEK2LRQTDVKAGH
FG07443.1 	LENTVKDFKKAADEGQQHGTQNETLQRRLQLLEEEAEDADKTLREANEK2LRQTDVKAGH
AN5686.1  	LESSAKEAKDAASQSAQHDTQNEALQRRIQLLEEEAEEADRNLRETNEK2LRQTDVKAGH
          	**  .*: *. *.:. . .**** * **:****:***:**:.***:*** **********

MG08078.1 	FERKVQALEAERDSWETKYEEMAKKYASTQKELEDLLAEVGNI
NCU01204.1	FERKVQALENERDQWESKYEEMAKKYAEVQKSLEEFQADIANI
FG07443.1 	FERKVQALESERDQWETKYEEMSQKYTSLQKELEELQAEIGNI
AN5686.1  	YERKVQALETSRDEWERKYEEMAAKHESLKKELAELELSLNSV
          	:******** .**.** *****: *: . :*.* ::  .: .:
```
